# Beyond the Foam Cell: The Role of LXRs in Preventing Atherogenesis

**DOI:** 10.3390/ijms19082307

**Published:** 2018-08-07

**Authors:** Adil Rasheed, Carolyn L. Cummins

**Affiliations:** 1 Department of Pharmaceutical Sciences, University of Toronto, Toronto, ON M5S 3M2, Canada; adil.rasheed@utoronto.ca; 2 Banting and Best Diabetes Centre, University of Toronto, Toronto, ON M5G 2C4, Canada; 3 The Heart and Stroke Richard Lewar Centre of Excellence in Cardiovascular Research, University of Toronto, Toronto, ON, M5S 3H2, Canada

**Keywords:** liver X receptors, atherosclerosis, cholesterol efflux, inflammation, macrophage, hematopoiesis, hematopoietic stem cells, neutrophils, endothelial cells, smooth muscle cells

## Abstract

Atherosclerosis is a chronic condition associated with cardiovascular disease. While largely identified by the accumulation of lipid-laden foam cells within the aorta later on in life, atherosclerosis develops over several stages and decades. During atherogenesis, various cell types of the aorta acquire a pro-inflammatory phenotype that initiates the cascade of signaling events facilitating the formation of these foam cells. The liver X receptors (LXRs) are nuclear receptors that upon activation induce the expression of transporters responsible for promoting cholesterol efflux. In addition to promoting cholesterol removal from the arterial wall, LXRs have potent anti-inflammatory actions via the transcriptional repression of key pro-inflammatory cytokines. These beneficial functions sparked an interest in the potential to target LXRs and the development of agonists as anti-atherogenic agents. These early studies focused on mediating the contributions of macrophages to the underlying pathogenesis. However, further evidence has since demonstrated that LXRs reduce atherosclerosis through their actions in multiple cell types apart from those monocytes/macrophages that infiltrate the lesion. LXRs and their target genes have profound effects on multiple other cells types of the hematopoietic system. Furthermore, LXRs can also mediate dysfunction within vascular cell types of the aorta including endothelial and smooth muscle cells. Taken together, these studies demonstrate the whole-body benefits of LXR activation with respect to anti-atherogenesis, and that LXRs remain a viable target for the treatment of atherosclerosis, with a reach which extends beyond plaque macrophages.

## 1. Introduction

Despite recent advances in drug development, cardiovascular disease (CVD) remains the number one cause of mortality worldwide [1]. Atherosclerosis, a chronic inflammatory complication associated with CVD, is characterized by the deposition of cholesterol-rich plaques that gradually narrow the aorta and decrease coronary blood flow over a lifetime [2,3]. During development, the growing atheroma remains stable; however, during advancement of the disease, the plaque becomes increasingly vulnerable. The terminal stages of atherosclerosis occur when the plaque ruptures, which can lead to vessel occlusion and thrombosis. Myocardial infarction or stroke resulting from plaque rupture contributes to almost 75% of CVD related deaths [3,4]. The contribution of atherosclerosis to global mortality underlies the need for novel therapeutics to prevent and/or reduce the plaque burden in CVD patients.

Studies from the early 2000s identified the liver X receptors (LXRα/β) as having potent anti-atherogenic functions. The anti-atherogenic function was attributed to their roles in macrophages to inhibit inflammation and promote reverse cholesterol transport. However, around this same period, LXRs were also identified as inducers of lipogenesis, where agonist treatment resulted in the development of a fatty liver [5]. This was attributed to the induction of the newly identified LXR target genes involved in fatty acid synthesis: acetyl-CoA carboxylase (ACC), fatty acid synthase (FAS), and stearoyl-CoA desaturase (SCD-1); as well as the master regulator of lipogenesis, sterol regulatory element binding factor 1c (SREBP-1c) [5,6,7]. These findings severely tempered the excitement surrounding LXRs as potential therapeutics for CVD. However, recent studies have demonstrated beneficial effects of LXRs in additional cell types, apart from the plaque macrophages, involved in atherogenesis. This, coupled with the continued development of next generation LXR agonists that circumvent the lipogenic toxicity, has provided renewed interest in the therapeutic targeting of LXRs. The pharmacologic strategies used to develop safer LXR agonists include generating compounds with tissue specific-, LXR isoform specific- and/or partial agonist activity and this aspect of LXR pharmacology has been reviewed elsewhere [8,9]. In this review, we will highlight new findings on the molecular role of LXRs in other hematopoietic and vascular cell types that contribute to its anti-atherosclerotic effects.

## 2. Pathogenesis of Atherosclerosis

### 2.1. Structure of the Aorta

The aorta is comprised of three well defined concentric layers. Beginning at the lumen, the first is the intimal layer, in which endothelial cells are seeded on a basement membrane. The middle or medial layer consists of vascular smooth muscle cells (SMCs) and basement membrane proteins. The final layer, the adventitial layer, is comprised of connective tissue and nerve fibers [10]. Endothelial and SMCs are involved the homeostatic function of the aorta, but also actively participate in atherogenesis. The following sections will detail the contributions of these two cell types and their interactions that facilitate the development of plaques in the aorta.

### 2.2. Contributions of the Endothelium to Atherosclerosis

The aortic endothelial cells form a physical barrier between the circulation and vasculature. These cells exist largely within a quiescent state, with a life span of over a year; however, they are also involved in active processes [11]. To repair damage, adjacent endothelial cells proliferate to preserve the integrity of the endothelium [2]. Under homeostatic conditions the endothelium has been exquisitely designed to maintain vascular integrity and undergo damage repair in response to physical and circulating factors by expressing antithrombotic and anti-inflammatory factors [12]. During the early stages of atherosclerosis, defects to the endothelium allow for adhesion and infiltration of circulating leukocytes. These types of lesions are characterized as Stage I–III [13]. These changes are broadly categorized as either: endothelial dysfunction, which includes a reduction in nitric oxide production and an increase in endothelial permeability; or endothelial activation, where endothelial cells adopt a pro-inflammatory phenotype [3].

#### 2.2.1. Endothelial Dysfunction

The prominent feature of endothelial dysfunction is an inability of the vessel to dilate due to a deficiency in nitric oxide production by endothelial nitric oxide synthase (eNOS). During atherogenesis, eNOS function is perturbed leading to decreased production of nitric oxide, or even a production of oxidative species that further potentiate defects to the endothelium, which can occur through nitric oxide scavenging and conversion to the pathogenic peroxynitrite species [14,15]. High shear stress and laminar blood flow induces mechanical stimulation of the eNOS enzyme in endothelial cells, whereas disturbed or low flow decreases eNOS activity [16,17]. Not surprisingly, given the role of the endothelium in initiating atherogenesis, areas of disturbed flow typically found at sites of arterial branching are correlated with increases in lipid deposition and plaque formation [11,18,19]. Low shear stress can also induce the release of the potent vasoconstrictor endothelin-1 and increase proteoglycan expression on endothelial cells, which in turn enhances binding of low-density lipoprotein (LDL) and promotes its uptake into the intima [20]. These defects to the endothelium are associated with the early stages of atherosclerosis. In the presence of an established plaque at the later stages of atherosclerosis, vasoconstriction and the inability of the vessel to dilate can lead to disruption and eventual rupture of the aortic plaque [11].

Another source of endothelial dysfunction manifests as a defect in endothelial cell turnover. Undisturbed laminar flow promotes the maintenance of endothelial quiescence via expression of key flow-dependent transcriptional regulators [21]. In contrast, areas exposed to turbulent flow are associated with impaired endothelial turnover and senescence [21,22]. Depending on the type of blood flow at the site of hyperlipidemic injury, alterations in endothelial cell turnover can promote atherosclerotic lesion formation [23].

#### 2.2.2. Endothelial Activation

In an atherosclerotic environment, leukocyte rolling is slowed and this promotes increased adhesion to the activated endothelium. Rolling is mediated by p- and e-selectin on the endothelial cells, which bind to sialyl-Lewis x or other carbohydrate moieties on the leukocytes [24]. Upon rolling, integrins on activated monocytes undergo conformational changes that increase the affinity for their respective adhesion molecules, such as vascular cell adhesion molecule 1 (VCAM-1) and intercellular adhesion molecule 1 (ICAM-1) [3]. Expression of these endothelial adhesion molecules are increased in the presence of oxidized LDL (oxLDL), tumor necrosis factor α (TNFα), and oxidative stress [3,14,25]. While both VCAM-1 and ICAM-1 are increased on activated endothelial cells, VCAM-1 expression has been correlated with the sites of plaque formation, whereas ICAM-1 is reported to strengthen the adhesion of monocytes through its interaction with integrins on the monocytes, but is not essential for plaque formation [26,27,28]. Increased adhesion of these leukocytes typically occurs in areas of low shear stress which are most vulnerable to plaque formation [29]. Low shear stress activates the key inflammatory transcription factor nuclear factor κB (NFκB) within endothelial cells, which in turn upregulates the expression of selectin and adhesion molecules (VCAM-1, ICAM-1, e-selectin) [30]. A key example of this is local production of the chemokine monocyte chemoattractant protein (MCP)-1 during atherogenesis by aortic endothelial cells, which coordinates the homing of monocytes [3,31]. Macrophage colony stimulating factor (M-CSF) expressed by the endothelium then allows for the differentiation of the recruited monocytes in the intima into macrophages [3].

Endothelial dysfunction and activation, however, do not occur as separate processes. Nitric oxide, aside from its effects on controlling vessel diameter, represses NFκB-dependent transcription of adhesion molecules, selectins, and inflammatory factors [32,33,34,35]. Endothelium-derived nitric oxide also prevents the modification of lipoproteins including oxidation of LDL, which has been demonstrated to be a major contributor to atherosclerotic plaque formation [3,36].

### 2.3. Late Stages of Atherosclerosis

Stage IV lesions are characterized by sequestration of extracellular lipids, particularly oxLDL, in macrophages and the subsequent development of foam cells within the intima forming the lipid core [37]. These macrophages produce pro-inflammatory factors that further potentiate endothelial cell defects and leukocyte entry into the intima, as well as increase inducible nitric oxide synthase (iNOS)–dependent production of nitric oxide and contributes to the oxidation of LDL, providing positive feedback to enhance atherogenesis [38,39].

During the advanced stages of atherogenesis, there is increased proliferation and migration of SMCs from the medial to intimal layer [10]. These changes are in part mediated by increases in endothelin-1 and decreases in nitric oxide by the dysfunctional endothelium, along with production of interleukin 1 (IL-1) by macrophages [15,40,41,42]. Furthermore, SMCs deviate from their homeostatic role in regulating vascular tone, to acquiring a synthetic state in which they release extracellular matrix proteins (i.e., collagens and elastins) [10,43,44]. The presence of this fibrous cap, comprised of SMCs and extracellular matrix proteins, is a key characteristic of Stage V lesions [37]. Recent studies have demonstrated that SMCs, like macrophages, uptake lipids and transdifferentiate into foam cells in the plaque [45,46]. A key difference is that SMC-derived foam cells have a reduced expression of the cholesterol efflux transporter ABCA1 compared to the macrophage-derived foam cells [47].

As atherosclerosis progresses, macrophages that take up lipids die, which in turn perpetuates the inflammatory nature of the plaque. Normally these apoptotic macrophages are cleared by efferocytosis; however, this process is defective during atherosclerosis. Apoptotic macrophages that cannot be cleared undergo secondary necrosis, rupture, and release their intracellular contents. This is the necrotic core of the plaque which can be formed from the physical disruption of the cell (necrosis) or cell-signaling mediated cellular release (necroptosis) [48]. Nonetheless, this process increases plaque vulnerability, yielding an acellular lesion of lipids (cholesterol clefts), which becomes less stable [49,50]. In its terminal stages (Stage VI lesions), the fibrous cap that once provided plaque stability thins by proteasomal degradation, particularly from the family of matrix metalloproteases (MMPs), elastases, and collagenases that are released from the intimal macrophages [10,51,52]. Recruitment and aggregation of platelets to the subendothelial plaque facilitates thrombus formation [49]. Plaque rupture releases the contents of the plaque, namely the accumulated lipids and inflammatory mediators which initiates a pro-coagulatory pathway, leading to stroke or myocardial infarction [53,54].

## 3. Liver X Receptors

The LXRs belong to the nuclear receptor superfamily of ligand activated transcription factors. Present in two isoforms, LXRβ (NR1H2) was first cloned in 1994, followed by LXRα (NR1H3) shortly thereafter [55,56,57,58,59,60]. While LXRβ is ubiquitously expressed, LXRα expression is highest in the liver, kidney, intestine, and adrenal glands [60,61,62]. The two isoforms share 77% sequence homology in their ligand binding domain [63]. Oxysterols, such as 27-hydroxycholesterol or 24(*S*),25-epoxycholesterol, are the endogenous ligands of LXRs [64,65]. The two LXR isoforms have largely overlapping functions, and in many cases, are able to compensate for the absence of one another [66]. LXRs are responsible for regulating multiple key metabolic pathways, such as coordinating cholesterol homeostasis, repressing inflammation, promoting lipogenesis, and many others. Because of these roles, LXRs represent a critical link between cholesterol homeostasis and immunity.

### 3.1. LXRs Preserve Cholesterol Homeostasis

The importance of LXRs in maintaining cholesterol homeostasis was observed when LXRα-knockout mice were fed a high cholesterol diet and developed blanched, enlarged livers rich in cholesteryl esters. LXRβ-knockout mice were protected against the development of this hepatic abnormality, highlighting the preferential basal role of LXRα in preserving liver cholesterol homeostasis [67,68]. In the periphery, activation of LXRs induce the expression of the ATP binding cassette (ABC) cholesterol efflux transporters *Abca1* and *Abcg1*, which facilitate efflux of free cholesterol from peripheral sites to lipid deficient apolipoprotein (Apo) A1 and high-density lipoprotein (HDL) particles, respectively [69,70,71,72]. LXRs also enhance the expression of *Apoe* and *Apoc* (*I*,*II*,*IV*), which are apolipoproteins that can be secreted by macrophages to promote cholesterol efflux to HDL [73,74]. LXRs also target two half transporters, *Abcg5* and *Abcg8*, that together form a functional protein which decreases the absorption of dietary cholesterol in the intestine and in the liver promotes the excretion of cholesterol to the bile [75,76,77]. Furthermore, LXRs decrease intestinal cholesterol absorption by decreasing expression of the Niemann-pick C1-like 1 (*Npc1l1*) transporter [78]. Indeed, LXR agonist treatment of mice promoted the efflux of radiolabeled cholesterol from macrophages to the feces via reverse cholesterol transport [79,80]. LXRα has also been reported to reduce cholesterol synthesis by repressing key biosynthetic enzymes lanosterol 14α-demethylase and squalene synthase in a human hepatic cell line [81].

### 3.2. LXRs Repress Inflammation

LXR monomers tether to key inflammatory transcription factors such as NFκB and AP-1, thereby repressing the transcription of their pro-inflammatory cytokine target genes such as *Il1b* and *Il6*, *Tnfa*, *Mcp1*, and *Inos* [82,83,84]. Repression of pro-inflammatory cytokines, such as *Inos* and *Il1b*, occurs through SUMOylation of the LXR monomer which prevents dissociation of the corepressor complex and thus transcription [85]. Furthermore, LXRs have been demonstrated to protect immune cells from pathogen-induced apoptosis (*E. coli*, *B. anthracis*, and *S. typhimurium*) through upregulation of anti-apoptotic factors and downregulation of pro-apoptotic factors [86]. These functions of LXRs have been primarily studied in macrophages. However, topical treatment with LXR agonists reduced inflammation at the site of induced contact dermatitis, implicating LXRs in repressing inflammation in non-macrophage populations as well [82,87]. LXRβ has also been found to repress the production of key inflammatory cytokines in stimulated mast cells, such as *Il1a* and *Il1b* [88].

## 4. LXRs and Atherosclerosis: A Macrophage Cholesterol Efflux-Centered Paradigm

The atheroprotective roles of LXRs have been studied using LXR gain- and loss-of-function models in atherosclerosis-prone *Apoe*^−/−^ and *Ldlr*^−/−^ mice (Table 1). Initial studies found that systemic administration of the LXR agonist GW3965 to either *Apoe*^−/−^ or *Ldlr*^−/−^ mice significantly decreased lesion formation [89]. Crosses of the LXR single isoform knockout mice to these atherosclerotic prone mouse backgrounds were subsequently employed to determine the relative contribution of each LXR isoform individually. Despite the seemingly redundant functions of LXRα and LXRβ in promoting cholesterol efflux after ligand activation, only the LXRα knockout (*Lxrα*^−/−^) mice bred to the *Apoe*^−/−^ or *Ldlr*^−/−^ backgrounds increased aortic lesions, which suggested that basally LXRβ was unable to compensate for the loss of LXRα to help prevent atherosclerosis [90,91].

In the studies detailed above, the mice were treated systemically with LXR agonists or LXRs were genetically deleted from all tissues. To test whether the anti-atherogenic effects of LXRs occurred through their role in macrophages, the Schulman group performed bone marrow transplant studies from *Lxrαβ*^−/−^ mice to *Apoe*^−/−^ and *Ldlr*^−/−^ recipient mice, which resulted in increased plaque formation compared wildtype bone marrow transplant, effects which were attributed to increases in macrophage cholesterol content [92]. The importance of the bone marrow-derived LXR target genes and cholesterol efflux transporters *Abca1* and *Abcg1* in preventing lesion formation was also shown in bone marrow transplant experiments from mice deficient in these transporters [93]. Furthermore, treatment of *Ldlr*^−/−^ mice with the LXR agonist T0901317 showed no effect on reducing lesion formation when these mice were transplanted with *Lxrαβ*^−/−^ bone marrow, whereas lesions were reduced when the T0901317-treated *Ldlr*^−/−^ mice received either wildtype or *Ldlr*^−/−^ bone marrow [94]. These observations were confirmed within *Ldlr*−/− mice in which macrophages transgenically overexpressing *Lxrα* showed decreases in atherosclerosis [95]. Together, these initial studies suggested that LXRs elicit their anti-atherogenic effects through promoting cholesterol efflux from intimal macrophages, thus reducing atherosclerotic plaques.

However, the hypothesis that LXRs are atheroprotective primarily by promoting macrophage cholesterol removal and reverse cholesterol transport has recently been challenged. The Schulman group found that *liver* expression of LXRα was critical to promote cholesterol efflux from macrophages and enhance elimination of cholesterol from the body [96]. While cholesterol homeostasis was perturbed in liver-specific *Lxrα*^−/−^ mice, hepatic LXRα was not required for the LXR agonist-dependent decreases in lesion area. In fact, intestinal-specific (but not liver-specific) overexpression of LXRα was required for reverse cholesterol transport and decreases in atherosclerotic lesion area [97]. Furthermore, bone marrow transplant experiments using donor bone marrow lacking *Abca1* and *Abcg1* in the whole bone marrow or specifically in the myeloid cell types of the bone marrow, indicate that LXR-mediated attenuation of lesion formation may occur independently of cholesterol efflux [98,99], suggesting additional mechanisms by which LXRs can elicit their anti-atherogenic effects. One such mechanism may involve the increase in *Lxr*α mRNA that is observed in foam cells during plaque regression [100]. LXRs upregulate *Ccr7* in macrophages, allowing their emigration from the plaque and thereby promote macrophage clearance and plaque regression [101,102,103].

## 5. LXRs and Hematopoietic Cell Types

### 5.1. Contributions of Hematopoietic Cell Types to Atherosclerosis

The bone marrow is a major source for the development of patrolling immune cells which are critical for both innate and adaptive immunity. All circulating immune cells belong to the hematopoietic lineage and are derived from hematopoietic stem cells (HSCs), which differentiate into hierarchical progenitor cell populations that give rise to the mature circulating immune cells of the myeloid and lymphoid lineages [104,105].

A majority of the focus on atherogenesis revolves around monocyte/macrophage recruitment and their contributions to the developing atheroma. However, in addition to monocytes, neutrophils and lymphocytes are similarly recruited to the aorta. The circulating numbers of monocytes and neutrophils are positively correlated with the extent of lesion development [106]. While neutrophils are rarely detected in the atherosclerotic lesion, they contribute to pathogenesis through the release of granule proteins; such as cathelicidins which promote the adhesion of inflammatory monocytes to activated endothelial cells; and myeloperoxidase which promotes oxidation of LDL to the pathogenic oxLDL [107,108]. T-cell subsets can also play a role in atherogenesis by influencing macrophage polarization. Type 1 helper T-cells release cytokines that allow for the development of M1 or pro-inflammatory macrophages, whereas type 2 helper T-cells release cytokines that allow for the development M2 or anti-inflammatory macrophages [109]. In addition, to macrophages, monocytes that infiltrate the plaque can differentiate to dendritic cells. These dendritic cells develop as part of the innate immune system and are antigen presenting cells that activate the adaptive immune system, primarily T-cells within the plaque. This in turn perpetuates the chronic inflammatory condition that underlies atherosclerosis [110].

### 5.2. LXRs and Their Target Genes Regulate Hematopoietic Cell Types: Implications for Atherosclerosis

The Tall group has extensively described the protective role of the LXR target genes *Abca1*, *Abcg1*, and *Apoe* in hematopoietic cell types, and their contributions to atherosclerosis [111,112]. They have eloquently shown using knockout mouse models (*Abca1*/*g1*^−/−^ and *Apoe*^−/−^) that lipid accumulation in HSCs, specifically cholesterol located in lipid rafts, leads to their proliferation. Excess cholesterol increases lipid raft signaling via the common β subunit of the Il-3 and granulocyte-monocyte colony stimulating factor (GM-CSF) receptors [111,112]. This, in turn, increases the numbers of the common myeloid and granulocyte-monocyte progenitors, which differentiate to monocytes and exacerbate the development of atherosclerosis [111,112]. HSC proliferation and formation of granulocyte and monocyte populations from excess lipid raft signaling was reversed by addition of HDL or ApoA1 [111,112,113]. Interestingly, HSC proliferation has also been noted in subjects with familial hypercholesterolemia and low HDL cholesterol levels [114]. Our group has recently reported an expansion of HSCs and myeloid progenitors in the bone marrow of chow-fed LXR-knockout mice, with increases in circulating monocytes and neutrophils [115]. Conversely, GW3965-treated HSCs isolated from wildtype but not LXR-null mice decreased myeloid colony formation [115]. These observations are in line with the studies performed using knockout *Abca1* and *Abcg1*, highlighting the role of LXR as a major upstream player in preserving hematopoiesis under pathological conditions.

Aside from regulating cholesterol efflux, knockout of *Abca1* and *Abcg1* also increases macrophage apoptosis induced by assembly of the NADPH oxidase 2 (Nox2) complex [116]. Macrophage-selective loss of these transporters increased the levels of circulating inflammatory cells, such as monocytes and neutrophils, due to increased macrophage secretion of M-CSF and granulocyte colony stimulating factor (G-CSF) [98]. LXR-dependent repression of *Mmp-9* within macrophages prevents the advancement of atherosclerosis through promoting plaque stabilization by preventing degradation of the fibrous cap [82,117]. In addition, expression of the LXR target gene *Cd5l*/*Spα* reduces plaque vulnerability by preventing macrophage apoptosis within the plaque [118]. LXR activation in macrophages upregulates the expression of the direct target gene vascular endothelial growth factor (*Vegf*) which is a known potent growth factor involved in preserving endothelial integrity [119]. Furthermore, LXRs influence macrophage polarization, by increasing the expression of the M2 (anti-inflammatory) macrophage markers and decreasing the expression of the M1 (pro-inflammatory) macrophage markers [82,120,121,122,123]. Together these studies demonstrate the importance of LXRs in preventing the advancement of the disease via a variety of mechanisms within macrophages.

Comparing the results of bone marrow transplants from *Abca1* and *Abcg1* double knockout mice versus macrophage-specific knockouts of *Abca1* and *Abcg1*, the Tall group found that knockout of these cholesterol efflux transporters in the whole bone marrow resulted in larger lesion area compared to bone from macrophage-specific knockouts [98]. Others have also demonstrated that myeloid specific-knockout of *Abca1* only had a marginal effect on lesion size in *Ldlr*^−/−^ mice when the mice were fed an atherogenic diet [124]. Together these studies suggest that bone marrow-derived cell types that are distinct from the monocyte/macrophage population also contribute to atherogenesis.

The Bensinger group has demonstrated the key role for LXRs in the phagocytosis of aged neutrophils. Activation of LXRs upregulates the expression of its target gene *Mertk*, which is the receptor responsible for facilitating the phagocytosis of apoptotic neutrophils. Furthermore, activation of LXRs represses Il-23 expression and release from macrophages, which is normally responsible for upregulating Il-17 release by T-cells leading to increased G-CSF expression in the bone marrow, thus facilitating granulopoiesis [125]. Additionally, phagocytosis of aged neutrophils by macrophages in the bone marrow decreases SDF-1α expression in the bone marrow in an LXR-dependent manner, which stimulates HSC differentiation and egress from the bone marrow [126]. Together these studies demonstrate that LXRs repress the production of key cell types (i.e., neutrophils) and processes (i.e., resolution of apoptotic cells) known to promote atherogenesis.

Recently, Beceiro et al. demonstrated a role for LXRs in dendritic cells. LXR activates the expression of cluster of differentiation 38 (CD38), an ectoenzyme critical for leukocyte chemotaxis. Dendritic cells isolated from LXR-null mice are deficient in stimulus-induced migration. Bone marrow transplant experiments using bone marrow from CD38 deficient donors had decreased lesion area compared to WT mice, implicating CD38 key regulator of myeloid cell migration and infiltration into the atherosclerotic plaque [127].

## 6. LXRs and Vascular Cell Types

The anti-atherogenic role of the LXRs, specifically LXRα, has also been demonstrated in non-hematopoietic cell types. This was demonstrated using bone marrow transplants of control bone marrow into *Ldlr*^−/−^ *Lxrα*^−/−^ mice which showed greater atherosclerotic plaque development than *Ldlr*^−/−^ mice receiving control bone marrow [91]. As previously described, defects in the aortic endothelium and SMCs contribute to atherogenesis. LXRs and their target genes have been demonstrated to elicit beneficial effects in both of these vascular cell types in addition to their known benefits on the hematopoietic system.

### 6.1. Endothelial Cells

Hemodynamic changes that occur during the development of atherosclerosis, including low sheer stress and turbulent flow, alter the expression of both LXR isoforms and their target genes Abca1 and Abcg1 [128]. Interestingly, expression of *Lxrα* and *Abca1* is 5-fold higher in the thoracic aorta, a region more resistant to the development of atherosclerotic lesions, as opposed to the aortic root, a region more atherosclerosis-prone [128]. Furthermore, shear stress on endothelial cells in culture induces the expression of the transcription factor Krüppel-like factor 4 (KLF4), which in turn upregulates the expression of 25-hydroxylase and LXRα [129]. T0901317 treatment of human aortic endothelial cells showed increases in SCD-1 which reduced palmitate-induced apoptosis and expression of pro-inflammatory cytokines [130]. Treatment of activated endothelial cells in culture with T0901317 reduced the expression of adhesion molecules (VCAM-1 and ICAM-1), angiotensin II receptors and response to the vasoconstrictive factor angiotensin II, apoptosis, and oxidative stress; and increased nitric oxide production through an increase in eNOS expression. These effects are attributed to LXRβ signaling in the endothelial cells [131,132]. In fact, LXRβ binding to the estrogen receptor α at the plasma membrane caveolae in endothelial cells increases eNOS activation and promotes re-endothelialization in vivo [133]. However, LXRα has also been demonstrated to have beneficial anti-inflammatory actions in activated endothelial cells in vitro by reducing sphingosine 1 phosphate receptor 2 expression and thus endothelial permeability through a mechanism involving miR-130a-3p upregulation [134]. Furthermore, GW3965 treatment of stimulated endothelial cells in vitro suppressed Il-8 expression via repression of NFκB dependent transcription, which in turn reduced monocyte-endothelial adherence, a key step in atherogenesis [135]. In a rat model of diabetic atherosclerosis, T0901317 was found to inhibit atherosclerosis and specifically endothelial cell senescence in part by induction of eNOS and inhibition of reactive oxygen species [136].

In models of plaque progression and plaque regression, T0901317 treatment reduced the endothelial expression of e-selectin, ICAM-1, and CD44; factors required for monocyte binding to the endothelium [101]. Furthermore, lipopolysaccharide-treated mice pretreated with GW3965 did not show increases in plasma endothelin-1, a vasoconstrictive factor produced by endothelial cells [137]. Loss of Abcg1 in endothelial cells was shown to accelerate mechanisms of atherosclerosis, such as increased monocyte-endothelial cell adhesion [138], and in the presence of HDL, eNOS uncoupling and nitric oxide synthesis through interactions of eNOS and caveolin-1 at the membrane [139,140]. Endothelial-specific knockouts of the LXR target genes *Abca1* and *Abcg1* show increased expression of pro-inflammatory cytokines and adhesion molecules in the aortic endothelium, promoting monocyte adhesion and development of atherosclerotic lesions [141]. Together these data indicate that LXRs can exert their anti-atherogenic effects through endothelial cell regulation of vessel diameter and leukocyte infiltration.

### 6.2. Smooth Muscle Cells

LXRs can also influence SMC-mediated contributions to atherogenesis although its role here has not been investigated as extensively as in other cell types. Like the function of LXRs in macrophages, LXR activation in SMCs induces expression of the cholesterol efflux transporters, while repressing the expression of pro-inflammatory cytokines [142]. Treatment with LXR agonist, decreased smooth muscle cell proliferation, neointima formation after carotid artery balloon injury, and decreased hypertension in response to angiotensin II [143,144]. While not directly regulating smooth muscle cells, LXR-mediated repression of NFκB-induced expression of Mmp-9, reduces degradation of the extracellular matrix secreted by smooth muscle cells that allow for plaque stability in later stage atherosclerosis [117].

## 7. Emerging Mechanisms of LXRs in Atherosclerosis

Beyond their roles as classical transcription factors, LXRs have recently been discovered to induce the expression of long non-coding RNAs (lncRNAs). The first of these LXR-regulated lncRNAs described was induced in the liver and named LeXis [145]. Overexpression of LeXis significantly decreased circulating plasma cholesterol. This effect was attributed to downregulation of hepatic cholesterol production by LeXis. In this context, LeXis interferes with a co-activator and RNA-binding protein, RALY, for the transcription of genes in the cholesterol biosynthetic pathway. A second long non-coding RNA, MeXis, is a macrophage-selective LXRβ-induced lncRNA. Bone marrow transplant of *Ldlr*^−/−^ mice with *MeXis*^−/−^ bone marrow demonstrated significantly increased atherosclerosis compared to WT bone marrow. MeXis expression enhances macrophage expression of Abca1 and subsequent cholesterol efflux [146]. By binding to the co-activator DEAD-box helicase 17 (DDX17), MeXis helps to direct co-activator binding to the Abca1 promoter and enhance transcription [146]. 

Aside from ligand-mediated activation, post-transcriptional regulation of LXRs is also a promising avenue for decreasing atherosclerosis. Tetratricopeptide repeat domain protein 39B (TTC39B) reduces hepatic LXRα protein expression through enhanced ubiquitination and proteasomal degradation [147]. In TTC39B-deficient mice, HDL cholesterol levels were increased, while the mice were protected from steatohepatitis, hepatic lipid synthesis, and cholesterol absorption compared to WT mice. Furthermore, when crossed with atherosclerotic *Ldlr*^−/−^ mice, *Ttc39b* knockout mice decreased atherosclerosis. These data demonstrate that by regulating hepatic LXR expression, as opposed to its activation, the beneficial effects associated with decreased atherosclerosis remain intact, while preventing the negative effects associated with the development of fatty liver.

## 8. Concluding Remarks

Since their discovery, LXRs have been investigated as potential therapeutics for the treatment of atherosclerosis. This was largely based on initial observations that LXRs promote cholesterol efflux and clearance from the vessel wall thus reducing the plaque burden associated with atherosclerosis, in addition to reducing inflammation. These functions were highly focused on the monocyte/macrophage populations. However, recent data using genetic deletion of LXRs or their target genes suggest that the anti-atherogenic roles of LXRs extent beyond macrophages. These studies have implicated a role of LXRs on the entire hematopoietic system, involving both production and clearance of various cell types. Aside from their regulation of hematopoietic cell types, LXRs also elicit beneficial effects on vascular cell types, including endothelial and smooth muscle cells. In parallel with the discovery of these new mechanisms of atheroprotection through LXR activation are the new strategies being employed to develop novel LXR agonists. Together, these recent advances in LXR research provide renewed promise to their role in decreasing atherosclerosis.

## Figures and Tables

**Table 1:** Key atherosclerosis experiments ^1^.

Reference	Description of Study ^2^		Major Findings	Conclusions
**Whole-body gain and loss-of-function studies**				
[148]	Chow-fed WT vs. *Lxrαβ*^−/−^ at 18 months		↑ lipid in aortic root of *Lxrαβ*^−/−^	LXRs regulate atherosclerotic development
[89]	WD-fed *Apoe*^−/−^, *Ldlr*^−/−^ ± GW (12 weeks)		↓ aortic root lesion area with GW	
[94]	WD-fed *Ldlr*^−/−^ ± T09 (oral gavage, 6 weeks)		T09: ↓ *en face* and aortic root lesion area, ↓ MΦ content, ↑ collagen content	
[101]	WD-fed Apoe*3Leiden Tg (18 weeks) + cholesterol-depleted diet ± T09 (8 weeks)		T09: ↓ aortic root lesion area, ↓ MΦ content	LXR activation promotes plaque regression
[102]	WD-fed *Ldlr*^−/−^ (6 weeks) + chow ± T09 (3 weeks)		T09: ↓ aortic root lesion area	
[90]	WD-fed *Apoe*^−/−^, *Lxrα*^−/−^*Apoe*^−/−^ ± GW (11 weeks)		↑ aortic root lesion area of *Lxrα*^−/−^*Apoe*^−/−^ compared to *Apoe*^−/−^	Basal LXRβ does not compensate for loss of LXRα w.r.t. lesion development; however, activation of LXRβ ↓atherosclerotic plaques in the absence of LXRα
			GW ↓ aortic root lesion area in *Apoe*^−/−^ and *Lxrα*^−/−^*Apoe*^−/−^	
[91]	WD-fed *Ldlr*^−/−^, *Lxrα*^−/−^*Ldlr*^−/−^, *Lxrβ*^−/−^ *Ldlr*^−/−^ ± T09 (12 weeks)		↑ aortic root lesion area in *Lxrα*^−/−^*Ldlr*^−/−^ vs. *Ldlr*^−/−^	
			T09: ↓ aortic root lesion area in *Ldlr*^−/−^, *Lxrα*^−/−^*Ldlr*^−/−^, and *Lxrβ*^−/−^ *Ldlr*^−/−^	
[147]	WD-fed *Ttc39b*^−/−^ vs. WT		*Ttc39b*^−/−^: ↓ steatohepatitis, cholesterol absorption, LXRα ubiquitination, ↑ HDL cholesterol	Increasing LXRα stability in the liver promotes its anti-atherogenic effects, while preventing negative effects associated with LXR activation
	WD-fed *Ttc39b*^−/−^ *Ldlr*^−/−^ vs. *Ldlr*^−/−^		↓ *en face* lesion area in *Ttc39b*^−/−^*Ldlr*^−/−^ vs. *Ldlr*^−/−^	
[136]	Sprague–Dawley rats + STZ ± T09		T09: ↓ *en face* lesion area, aortic intimal senescence	LXR decreases aortic endothelial cell senescence, decreasing atherosclerosis
**Bone marrow transplant studies ^3^**				
[92]	1) *Apoe*^−/−^ 2) WT 3) *Lxrαβ*^−/−^ 4) *Ldlr*^−/−^ 5) WT 6) *Lxrαβ*^−/−^	➔ *Apoe*^−/−^ ➔ *Apoe*^−/−^ ➔ *Apoe*^−/−^ ➔ *Ldlr*^−/−^ ➔ *Ldlr*^−/−^ ➔ *Ldlr*^−/−^	↑ *en face* lesion area in *Apoe*^−/−^ and *Ldlr*^−/−^ mice receiving *Lxrαβ*^−/−^ BM (*Apoe*^−/−^: 3 > 1 > 2; *Ldlr*^−/−^: 6 > 4,5)	Cholesterol efflux in macrophages is responsible for the LXR-mediated effects on reducing atherosclerotic lesions
[94]	1) *Ldlr*^−/−^ 2) WT 3) *Lxrαβ*^−/−^	➔ *Ldlr*^−/−^ ± T09 ➔ *Ldlr*^−/−^ ± T09 ➔ *Ldlr*^−/−^ ± T09	T09: ↓ *en face* lesion area from WT and *Ldlr*^−/−^ donors	
[102]	1) WT ➔ *Apoe*^−/−^ (3 days WD-early lesion) 2) WT ➔ *Apoe*^−/−^ (3 weeks WD-advanced lesion) After BMT, recipients switched to chow ± T09		T09: ↓ early and late aortic root lesion area, ↓ MΦ content	LXR activation promotes plaque regression
[103]	WD-fed *Apoe*^−/−^ ± T09		T09: ↓ aortic arch MΦ content	
	Aortic Arch transplants to WT mice after the following BMTs and 16 weeks WD:		↑ Aortic plaque lesion area and monocyte area of mice from *Lxrα*^−/−^ *Apoe*^−/−^ or *Lxrβ*^−/−^ *Apoe*^−/−^ donors (2, 3 > 1)	
	1) *Apoe*^−/−^ 2) *Lxrα*^−/−^*Apoe*^−/−^ 3) *Lxrβ*^−/−^*Apoe*^−/−^	➔ *Apoe*^−/−^ ➔ *Apoe*^−/−^ ➔ *Apoe*^−/−^		
[91]	1) *Ldlr*^−/−^ 2) *Lxrα*^−/−^*Ldlr*^−/−^ 3) *Ldlr*^−/−^ 4) *Lxrα*^−/−^*Ldlr*^−/−^	➔ *Ldlr*^−/−^ ➔ *Ldlr*^−/−^ ➔ *Lxrα*^−/−^*Ldlr*^−/−^ ➔ *Lxrα*^−/−^*Ldlr*^−/−^	↑ *en face* lesion area in 2 vs. 1 ↑ *en face* lesion area in 4 vs. 2 ↑ *en face* lesion area in 4 vs. 3 ↑ *en face* lesion area in 3 vs. 1	LXRα also has anti-atherogenic effects in non-hematopoietic cells (3 vs. 1)
[99]	1) WT 2) *Abca1*/*g1*^−/−^ 3) *Abca1*/*g1*^−/−^ 4) *Abca1*/*g1*^−/−(myeloid)^	➔ *Ldlr*^−/−^ ± T09 ➔ *Ldlr*^−/−^ ± T09 ➔ *Ldlr*^−/−^ ± GW ➔ *Ldlr*^−/−^ ± GW	T09: ↓ aortic root lesion area in 2 but not 1; ↓ inflammatory cell infiltration in 2 GW: ↓ aortic root lesion area 3 and 4; (greater ↓ in 3 vs. 4)	LXRs can mediate anti-atherogenic effects via BM cells independent of cholesterol efflux from myeloid cells Loss of LXR target genes in BM cells ↑ atherosclerosis Under chow feeding, *Abca1/g1*^−/−^ from whole BM ↑ lesion area relative to *Abca1/g1*^−/−^ only in myeloid cells
[98]	1) WT and *fl/fl* 2) *Abca1*/*g1*^−/−^ 3) *Abca1*/*g1*^−/−(myeloid)^	➔ *Ldlr*^−/−^ (chow) ➔ *Ldlr*^−/−^ (chow) ➔ *Ldlr*^−/−^ (chow)	Aortic root lesion area: 2 > 3 > 1	
[149]	1) WT 2) *Abca1*^−/−^ 3) *Apoe*^−/−^ 4) *Abca1*^−/−^*Apoe*^−/−^	➔ *Ldlr*^−/−^ ➔ *Ldlr*^−/−^ ➔ *Ldlr*^−/−^ ➔ *Ldlr*^−/−^	*En face* and aortic root lesion area: 4 > 3 = 2 > 1	
[124]	1) *Ldlr*^−/−^ 2) *Abca1*^−/−(myeloid)^ *Ldlr*^−/−^	➔ *Ldlr*^−/−^ ➔ *Ldlr*^−/−^	Aortic root lesion area: 2 > 1 (chow-fed); 2 = 1 (WD-fed)	
**Tissue-specific gain and loss-of-function studies**				
[95]	WD-fed *Lxrα*-Tg^(macrophage)^ *Ldlr*^−/−^ vs. *Ldlr*^−/−^		*Lxrα*-Tg^(macrophage)^*Ldlr*^−/−^: ↓ BCA lesion area, ↑ BMDM cholesterol efflux, ↓ BMDM nitric oxide production	Macrophage OE of LXRs ↓ lesion area
[96]	*Lxrα*^−/−(liver)^ vs. WT ± T09		*Lxrα*^−/−(liver)^: ↓ T09-induced increases in circulating triglycerides & cholesterol excretion *Lxrα*^−/−(liver)^: ↓ T09-induced decrease in intestinal cholesterol absorption	Hepatic LXRα is required for agonist-mediated RCT but not ↓ atherosclerosis, whereas intestinal LXRα OE does facilitate RCT and ↓ atherosclerosis
[96]	WD-fed *Lxrα*^−/−(liver)^ *Ldlr*^−/−^ vs. *Ldlr*^−/−^ ± T09		T09: ↓ *en face* lesion area in both genotypes	
[97]	WD-fed *Lxrα*-Tg^(intestine)^ vs. WT		*Lxrα*-Tg^(intestine)^: ↓ hepatic cholesterol & triglycerides, ↑ circulating VLDL triglycerides, ↑ HDL cholesterol	
[97]	WD-fed *Lxrα*-Tg^(intestine)^ *Ldlr*^−/−^ vs. *Ldlr*^−/−^		*Lxrα*-Tg ^(intestine)^*Ldlr*^−/−^: ↓ *en face* & aortic sinus lesion area	
[143]	Carotid artery injury in Sprague–Dawley rats ± T09		T09: ↓ neointimal formation	LXR target genes can also affect non-hematopoietic cells (i.e., endothelial, smooth muscle) to ↓ atherogenesis
[140]	1) WD-fed WT 2) WD-fed *Abca1*^−/−^ 3) WD-fed *Abcg1*^−/−^ 4) WD-fed *Abca1*^−/−^*Abcg1*^−/−^		Aortic eNOS-caveolin interaction: 4 > 3 > 2 > 1	
[138]	*Abcg1*^−/−^ vs. WT		*Abcg1*^−/−^: ↑ monocyte adherence to aortic endothelium	
[141]	*Abca1/g1*^−/−(endothelial)^*Ldlr*^−/−^ vs. *Ldlr*^−/−^		*Abca1/g1*^−/−(endothelial)^: ↑ *en face* and aortic root lesion area, ↑ MΦ content, ↑ monocyte recruitment, ↑ LPS-induced endothelial expression of pro-inflammatory and adhesion molecules	
**LncRNAs**				
[145]	Chow-fed Ad-LeXis vs. Ad-GFP		Ad-LeXis: ↓ plasma cholesterol, hepatic cholesterol biosynthetic gene expression	LncRNA targets of LXRs work to enhance cholesterol efflux and repress cholesterol synthesis, together enhancing the anti-atherogenic effects of LXRs
	WD-fed *LeXis*^−/−^ vs. WT		*LeXis*^−/−^: ↑ hepatic cholesterol	
[146]	BMT^3^: 1) WT ➔ *Ldlr*^−/−^ 2) *MeXis*^−/−^ ➔ *Ldlr*^−/−^		*MeXis*^−/−^: ↑ *en face* lesion area, ↓ aortic MΦ *Abca1*	

^1^ All experiments were performed in mice unless otherwise indicated. ^2^ Abbreviations used: ↑, increased; ↓, decreased; Ad, adenoviral; BCA, brachiocephalic artery; BM, bone marrow; BMDM, bone marrow-derived macrophages; BMT, bone marrow transplant; GW, GW3965; MΦ, Macrophage; OE, overexpression; RCT, reverse cholesterol transport; STZ, streptozotocin; T09, T0901317; Tg, transgenic; VLDL, very low-density lipoprotein; WD, Western diet; w.r.t., with respect to; WT, wildtype. ^3^ All BMT recipient mice fed a WD unless otherwise indicated.

## Abbreviations

CVD	Cardiovascular disease
LXR	Liver X receptor
SREBP-1c	Sterol response element binding protein-1c
ACC	Acetyl-CoA carboxylase
FAS	Fatty acid synthase
SCD-1	Stearoyl-CoA desaturase-1
SMC	Smooth muscle cell
eNOS	Endothelial nitric oxide synthase
LDL	Low-density lipoprotein
VLA-4	Very late antigen-4
VCAM-1	Vascular cell adhesion molecule-1
oxLDL	Oxidized low-density lipoprotein
TNFα	Tumor necrosis factor α
ICAM-1	Intercellular adhesion molecule-1
NFκB	Nuclear factor κB
M-CSF	Macrophage colony stimulating factor
iNOS	Inducible nitric oxide synthase
Il	Interleukin
MMP	Matrix metalloprotease
Apo	Apolipoprotein
HDL	High-density lipoprotein
Npc1l1	Niemann-pick C1-like 1
MCP-1	Monocyte chemoattractant protein-1
LDLR	Low-density lipoprotein receptor
CCR7	C-c chemokine receptor 7
HSC	Hematopoietic stem cell
GM-CSF	Granulocyte-monocyte colony stimulating factor
Nox2	NADPH oxidase 2
G-CSF	Granulocyte colony stimulating factor
VEGF	Vascular endothelial growth factor
CD38	Cluster of differentiation 38
KLF4	Krüppel-like factor 4
S1PR2	Sphingosine-1-phosphate receptor 2
LPS	Lipopolysaccharide
LncRNA	Long non-coding RNA
DDX17	DEAD-box helicase 17
TTC39B	Tetratricopeptide repeat domain protein 39B
WT	Wildtype
WD	Western diet
Tg	Transgenic
MΦ	Macrophage
T09	T0901317
GW	GW3965
STZ	Streptozotocin
BCA	Brachiocephalic artery
BM	Bone marrow
BMDM	Bone marrow-derived macrophages
BMT	Bone marrow transplant
OE	Overexpression
RCT	Reverse cholesterol transport
VLDL	Very low-density lipoprotein
